# Post-Dengue Parsonage-Turner Syndrome: A Case Report

**DOI:** 10.7759/cureus.69326

**Published:** 2024-09-13

**Authors:** Lorena Adolphsson, Billy McBenedict, Bruno Lima Pessôa, Thiago De Mello Tavares, Marco Orsini

**Affiliations:** 1 Neurosurgery, Fluminense Federal University, Niterói, BRA; 2 Family Medicine, Santo Amaro da Imperatriz Municipal Health Department, Santa Catarina, BRA; 3 Psychiatry, Federal University of Rio de Janeiro, Rio de Janeiro, BRA; 4 Health Surveillance, Iguaçu University, Nova Iguaçu, BRA

**Keywords:** dengue virus, electromyography, muscle atrophy, neuropathic pain, parsonage-turner syndrome

## Abstract

Parsonage-Turner syndrome (PTS) is a non-traumatic disorder of the shoulder girdle, marked by sudden-onset neuropathic pain, spontaneous improvement, and progression to paralysis, muscle weakness, and atrophy. Various etiological factors have been linked to PTS. This clinical case report documents the development of PTS following dengue virus exposure. Laboratory tests, electromyography, and imaging studies ruled out other potential diseases. This report highlights dengue infection as a potential infectious trigger for PTS and discusses its clinical presentation, treatment, and prognosis through a narrative review of the case presented.

## Introduction

Parsonage-Turner syndrome (PTS) is a non-traumatic condition affecting the shoulder girdle, characterized by sudden and intense neuropathic pain that typically lasts about two to three weeks and then spontaneously improves. This initial phase is followed by muscle weakness, paralysis, and atrophy corresponding to the affected nerve's distribution [1]. The syndrome was first mentioned in the 19th century, but it was not fully described until 1948 by Parsonage and Turner, who studied a series of 136 cases [2,3].

The etiology of Parsonage-Turner syndrome (PTS) remains unknown, but studies suggest the involvement of infectious and autoimmune factors. Approximately 25-55% of diagnosed patients have a history of viral infection or immunization [4] poxvirus, influenza virus, and coxsackievirus. Additionally, some bacteria have been associated with PTS cases, notably *Rickettsia prowazekii*, the causative agent of typhus, and *Borrelia burgdorferi*, the causative agent of Lyme disease [1,4]. Besides infectious and autoimmune factors, PTS can also manifest as an autosomal dominant hereditary condition known as hereditary neuralgic amyotrophy, which is characterized by recurrent episodes of peripheral nerve damage [2].

PTS is also a diagnosis of exclusion, necessitating the ruling out of other pathologies such as rotator cuff tear, adhesive capsulitis, cervical spondylopathy, and neurological disorders like peripheral nerve compression, acute poliomyelitis, and amyotrophic lateral sclerosis [1]. Consequently, further investigation is required, which includes laboratory tests, primarily a complete blood count with infectious, autoimmune, and inflammatory screening, electromyography, and imaging studies like magnetic resonance imaging (MRI).

PTS is a rare neurological condition with an occurrence rate of two to three cases per 100,000 people annually. However, the condition is often missed in diagnoses, leading experts to believe the true occurrence might be closer to 20-30 cases per 100,000 individuals each year [5,6]. PTS shows a higher prevalence in males compared to females, with male-to-female ratios ranging from 2:1 up to 11.5:1. This syndrome typically manifests in people aged between 30 and 40 years [1,4]. The outlook for those with PTS is generally positive, as the disorder tends to resolve on its own, and the likelihood of recurrence is low. Treatment strategies mainly focus on pain management and physical therapy to support muscle function and mobility [1].

Furthermore, as an underdiagnosed disease, it is essential to describe the probable etiologies in the literature to consider PTS among the differential diagnoses and increase awareness in the medical field. To this end, this case report aims to describe a rare or unknown cause of PTS by detailing its clinical presentation, treatment, and prognosis through a narrative review of the presented case.

## Case presentation

A 63-year-old retired female with a history of mild hypertension developed significant symptoms approximately 21 days after a dengue virus infection. She experienced severe pain primarily in the proximal and intermediate third of her right arm, accompanied by limb atrophy and skin color changes consistent with livedo. The pain, described as stabbing, shocks, and a burning sensation, became unbearable and had neuropathic characteristics. She was unable to find comfortable positions to change her decubitus and could not perform functional tasks. Her functional impairment was marked by a triad of pain, muscle weakness, and joint instability.

On neurological examination, she exhibited amyotrophy in the proximal and intermediate thirds of the right upper limb and hypotonia, with grade 2/3 paresis in the deltoid, infraspinatus, biceps brachii, brachioradialis, and triceps brachii muscles. Superficial sensitivity testing revealed allodynia to touch and pressure, along with thermal hyperesthesia and neuropathic pain, while deep sensitivity was characterized by hypoesthesia. Deep tendon reflexes of the biceps, triceps, and brachioradialis were absent on the right side. The cranial nerves were normal.

A complete workup was conducted to screen for inflammatory, infectious, and immune-mediated diseases. The results were normal except for a positive immunoglobulin M (IgM) for dengue virus. Additionally, electroneuromyography indicated acute denervation of peripheral nerves with prolonged action potentials and latency (Table 1). Electroneuromyography (ENMG) assessments, focused on both sensory and motor nerve function in the upper extremities. The sensory nerve tests included latency (Lat, ms), amplitude (Amp, μV), and conduction velocity (Vel, m/s) for various nerves, such as the median, ulnar, and radial nerves, tested at different points (e.g., III finger, V finger, I finger). The motor nerve test similarly detailed latency, amplitude, and velocity, specifically at the wrist (fist) and elbow, providing a comprehensive overview of nerve function. This data was crucial for diagnosing and understanding the extent of peripheral nerve involvement. 

**Table 1:** Electroneuromyography results of sensory and motor nerve assessments There is a significant reduction, especially in the sensory nerves on the right side, in both amplitude and velocity, along with an increase in the latency period. These findings indicate acute denervation of the peripheral nerves.

Sensory Nerves				Motor Nerves				
Nerve	Latency (ms)	Amplitude (μV)	Conduction Velocity (m/s)	Nerve		Latency (ms)	Amplitude (μV)	Conduction Velocity (m/s)
Left median nerve; III finger	3,3	24	56,5	Left ulnar; abductor minimus	Fist	2,5	8,3	-
Right median nerve; III finger	4,3	6,2	39,8		Elbow	6,2	7,3	61,2
Left ulnar nerve; V finger	2,9	22,8	63,6	Right ulnar; abductor minimus	Fist	2,8	4,4	-
Right ulnar nerve; V finger	3,3	6	54,7		Elbow	7,5	5,5	44,9
Left radial nerve; I finger	2,4	6,1	55,6	Left median; abductor pollicis brevis	Fist	3,6	10,6	-
Right radial nerve; I finger	3,7	2,4	32,1		Elbow	7,4	8,5	59,9
Left median nerve; I finger	3	15,5	43,9	Right median; abductor pollicis brevis	Fist	3,9	4,8	-
right median nerve; I finger	3,6	7,6	32,9		Elbow	8,6	5,8	46,4

**Figure 1:**
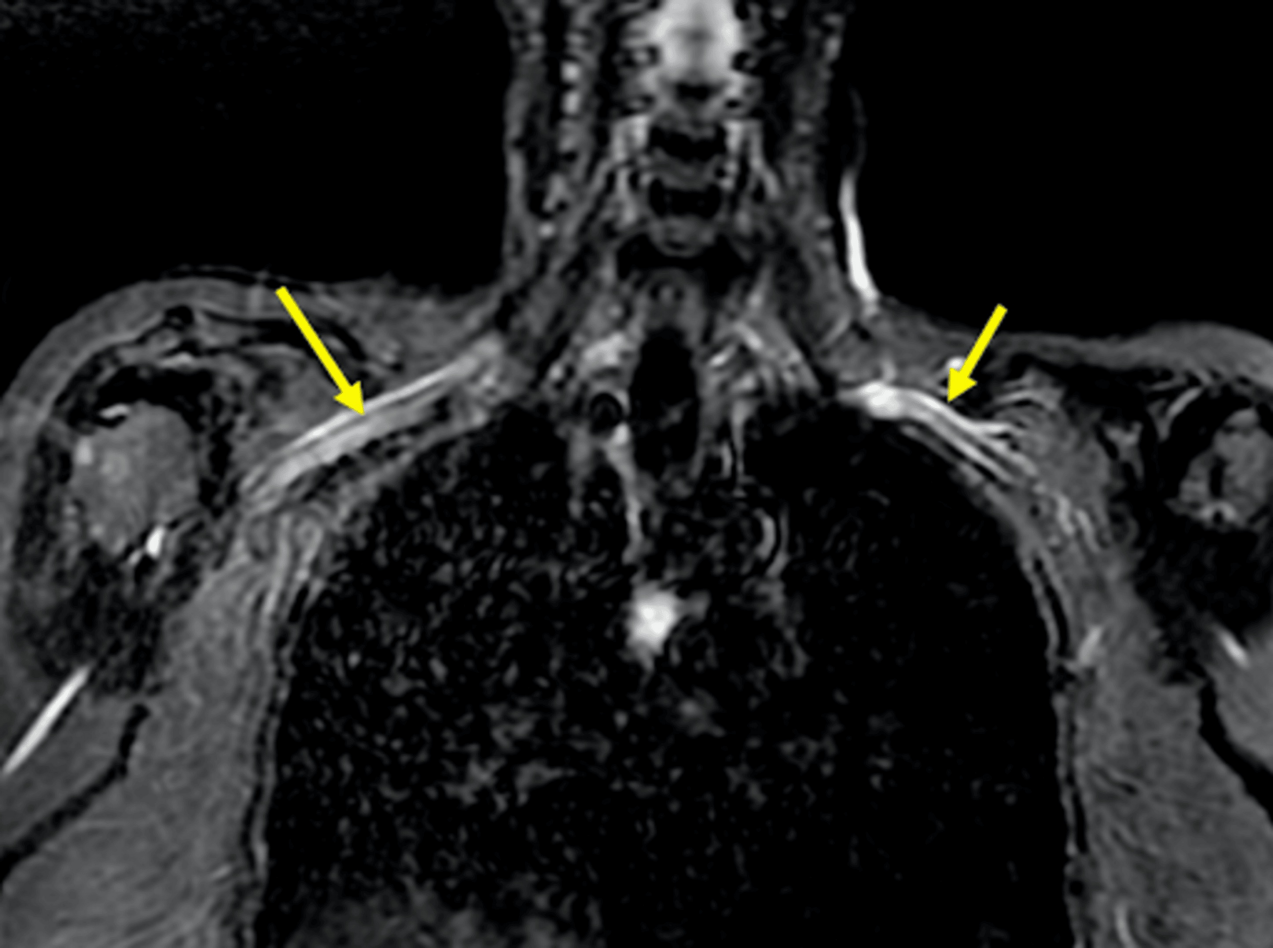
An MRI scan showing signs of brachial plexus neuritis with bilateral edema The yellow arrows indicate areas of bilateral edema, which are represented by a hypersignal on the short tau inversion recovery (STIR) sequence.

MRI of the brachial plexus revealed slight signal alterations in the upper and middle primary trunks (Figure 1). ENMG was performed to evaluate the etiology of the motor and sensory deficits, while MRI was used to better assess the presence and extent of inflammation. After evaluating numerous potential diagnoses, including rotator cuff injuries, calcific tendinitis, frozen shoulder, cervical spine disorders, nerve entrapments, brachial plexus compression by a cervical rib, post-polio sequelae, and amyotrophic lateral sclerosis, all other diagnostic avenues were ruled out. Consequently, it was determined that the patient most likely has PTS, which may have been precipitated by a recent dengue infection. Currently, the patient has been taking 60 mg of duloxetine, 300 mg of pregabalin, and 40 mg of cannabidiol. The pain has been significantly reduced, leading to an improvement in paresis. However, the patient continues to experience some difficulty with movements that require endurance and muscular strength.

## Discussion

Accurate diagnosis of PTS also referred to as idiopathic brachial plexopathy or neuralgic amyotrophy, is challenging and necessitates a comprehensive clinical evaluation, as it shares similarities with various other upper limb conditions, including rotator cuff disorders, nerve entrapments, cervical spine issues, acute calcific tendinitis, adhesive capsulitis, tumors, acute poliomyelitis, and amyotrophic lateral sclerosis [7].

PTS typically presents with a distinct pattern of intense pain followed by marked muscle weakness. The pain is often described as a severe, throbbing sensation that radiates from the shoulder, extending down the arm or upward into the neck [8]. The patient in the current case reported progressively worsening pain with neuropathic features such as stabbing, shock-like sensations, and a burning feeling. Additionally, she experienced functional limitations due to the combination of pain, muscle weakness, and joint instability. In PTS, muscle weakness generally appears after the acute pain phase, with around 70% of patients developing weakness approximately two weeks following the onset of symptoms [9]. Although this case involves a female patient, research indicates that PTS predominantly affects males, with the highest incidence observed in individuals during their third and seventh decades of life [10].

The onset of PTS typically follows a bell-shaped distribution, with the majority of cases occurring in middle-aged adults, while occurrences in children and the elderly are quite rare [11,12]. Magee and DeJong (1960) observed that there appears to be no correlation between PTS and hand dominance, though the condition is bilateral in approximately one-third of cases.

An important detail, in this case, is that the patient experienced severe pain in the proximal and middle thirds of the right arm approximately 21 days after contracting dengue virus infection. Infectious prodrome is recognized as the most common trigger for PTS [9]. Tsairis et al. (1972) reported that 25 out of 99 patients had a preceding flu-like illness before the onset of PTS. While there is no evidence of direct viral invasion of the nerves, it is believed that PTS is caused by a parainfectious inflammatory immune response. A wide range of infections has been associated with PTS, including COVID-19, cytomegalovirus, coxsackie B, diphtheria, dengue fever, herpes zoster, HIV, parvovirus, papillomavirus, tetanus, and typhoid [9].

In most cases, including the one presented here, the results of routine laboratory tests such as full blood count, blood biochemistry, immunoglobulin levels, and urinalysis are generally unremarkable. However, electromyography (EMG) often plays a key role in confirming the diagnosis of PTS. In this case, ENMG revealed signs of acute denervation in the peripheral nerves, characterized by prolonged action potentials and increased latency. Additionally, MRI of the brachial plexus showed subtle signal changes in the upper and middle trunks. These electromyographic abnormalities typically manifest approximately three weeks after the initial onset of symptoms [8]. MRI has recently emerged as a valuable tool in diagnosing brachial neuritis, and its effectiveness is expected to further improve with advancements in imaging resolution and quality [9].

PTS is generally a self-limiting disorder, with 80-90% of patients regaining muscle strength within two to three years. However, up to 70% of individuals may continue to experience residual weakness and exercise intolerance [13]. Treatment is primarily supportive, focusing on managing symptoms and facilitating recovery. Acute neuropathic pain in PTS is typically treated with long-acting opioids and nonsteroidal anti-inflammatory drugs (NSAIDs) like diclofenac. Physical therapy is essential for addressing musculoskeletal pain caused by compensatory muscle imbalances, helping to restore normal movement patterns [13, 8]. Tsairis et al. (1972) found that corticosteroids did not provide significant benefit to patients. Effective pain control during the early stages of the condition is crucial. While there is limited evidence supporting the use of therapeutic modalities such as massage, ultrasound, or electrical stimulation, these approaches may be considered adjuncts to pain management. The overall prognosis for PTS is favorable, with approximately 75% of patients achieving full recovery within two years [14]. Surgical intervention is reserved for those who fail to improve after an extended period. Although relapses can occur, they tend to be milder and shorter in duration.

## Conclusions

This case report emphasizes the importance of recognizing dengue virus infection as a potential trigger for PTS, a rare and often underdiagnosed disorder frequently associated with infections, surgeries, and immunizations. Highlighting dengue as a contributing factor to PTS deepens our understanding of the syndrome's implications and etiologies. PTS is a serious condition that, if not diagnosed and treated promptly, can lead to irreversible muscle atrophy. Accurate diagnosis requires a comprehensive clinical evaluation, including electromyography and imaging studies, to rule out other potential causes of brachial neuritis. Early recognition and intervention are crucial for effective management, focusing on pain relief and physical therapy to mitigate muscle atrophy and enhance recovery. Although PTS is generally self-limiting, with most patients experiencing gradual improvement, some may suffer from residual weakness and exercise intolerance. Timely intervention and supportive care are essential for a favorable prognosis. This case adds to the growing evidence that post-infectious inflammatory responses may significantly contribute to PTS, underscoring the need for future research to explore the pathophysiological mechanisms linking viral infections like dengue to this syndrome, potentially leading to more targeted therapeutic approaches.

## References

[1] Dos Santos RB, Dos Santos SM, Leal FJ, Lins OG, Magalhães C, Fittipaldi RB (2015). Parsonage-Turner syndrome [Article in Spanish]. Rev Bras Ortop.

[2] van Alfen N, van Engelen BG (2006). The clinical spectrum of neuralgic amyotrophy in 246 cases. Brain.

[3] Parsonage MJ, Turter JW (1948). Neuralgic amyotrophy; the shoulder-girdle syndrome. Lancet.

[4] Oliveira SG, Pombo EH, Batista PR, Cardoso IM, Rezende R (2010). Parsonage-Turner syndrome: case report of a HIV-seropositive patient. Rev Bras Ortop.

[5] Brites HG, Rosa FS, Gomes JS, Lima AV (2021). Parsonage turner syndrome: a case report. Arq Catarin Med.

[6] Advances in Neurology and in Your Clinical Practice 3. Adv Neurol Clin Prac.

[7] Misamore GW, Lehman DE (1996). Parsonage-turner syndrome (acute brachial neuritis). J Bone Jt Surg.

[8] Hussey AJ, O'Brien CP, Regan PJ (2007). Parsonage-Turner syndrome-case report and literature review. Hand (N Y).

[9] Meiling JB, Boon AJ, Niu Z, Howe BM, Hoskote SS, Spinner RJ, Klein CJ (2024). Parsonage-Turner syndrome and hereditary brachial plexus neuropathy. Mayo Clin Proc.

[10] Magee KR, DeJong RN (1960). Paralytic brachial neuritis. Discussion of clinical features with review of 23 cases. JAMA.

[11] Beghi E, Kurland LT, Mulder DW, Nicolosi A (1985). Brachial plexus neuropathy in the population of Rochester, Minnesota, 1970-1981. Ann Neurol.

[12] MacDonald BK, Cockerell OC, Sander JW, Shorvon SD (2000). The incidence and lifetime prevalence of neurological disorders in a prospective community-based study in the UK. Brain.

[13] Carrier RE, Marchetti MP (2022). Parsonage-Turner syndrome of unclear causation: a case report. Cureus.

[14] Tsairis P, Dyck PJ, Mulder DW (1972). Natural history of brachial plexus neuropathy. Report on 99 patients. Arch Neurol.

